# Transient architecture of the embryonic pancreas determines endocrine mass

**DOI:** 10.21203/rs.3.rs-10935206/v1

**Published:** 2026-09-14

**Authors:** Neha H. Ahuja, Christopher Chaney, Tyler Bierschenk, Tuli Pramanik, Austin Mills, Peter M. Luo, Mitzy A. Cowdin, Jinlong Lin, Jun Tsunezumi, Kevin M. Dean, Denise K. Marciano, Thomas J. Carroll, Ondine Cleaver

**Affiliations:** 1 Department of Molecular Biology, University of Texas Southwestern Medical Center, 5323 Harry Hines Blvd., Dallas, Texas, USA 75390; 2 Internal Medicine (Division of Nephrology), University of Texas Southwestern Medical Center, 5323 Harry Hines Blvd., Dallas, Texas, USA 75390; 3 Lyda Hill Department of Bioinformatics, University of Texas Southwestern Medical Center, 5323 Harry Hines Blvd., Dallas, Texas, USA 75390; 4 Cecil H. and Ida Green Center for Systems Biology, University of Texas Southwestern Medical Center, 5323 Harry Hines Blvd., Dallas, Texas, USA 75390; 5 Cell Biology, University of Texas Southwestern Medical Center, 5323 Harry Hines Blvd., Dallas, Texas, USA 75390

**Keywords:** Pancreas, organogenesis, epithelial architecture, plexus, lumenogenesis, lumen, endocrine mass, Merlin, *Nf2*, epithelium, morphogenesis, cell fate, vesicle trafficking, Rab5, Rab11, Crumbs3, PI3K, Hippo, Yap1, Ezrin

## Abstract

During organogenesis, epithelial tissues undergo extensive three-dimensional remodeling while simultaneously generating specialized cell types, yet whether transient architectural states actively instruct lineage allocation remains unclear. During pancreas development, *de novo* lumen formation transforms a stratified epithelium into a transient lumenal plexus from which endocrine, acinar and ductal lineages emerge. Here we identify the mechanosensitive Hippo pathway regulator Merlin as a critical link between epithelial architecture and cell fate. Loss of Merlin impairs *de novo* lumen formation, preventing epithelial destratification and establishment of the pancreatic plexus. Disruption of this architecture alters lineage allocation, impairing acinar differentiation and ultimately reducing adult endocrine cell mass and glucose homeostasis. Mosaic deletion reveals that these lineage defects arise non-cell autonomously, demonstrating that epithelial architecture itself instructs cell fate decisions. Mechanistically, Merlin coordinates polarized membrane trafficking to nascent lumens and restrains PI3K signaling; PI3K activation phenocopies Merlin loss, whereas PI3K inhibition partially rescues epithelial morphogenesis. Together, these findings establish the pancreatic plexus as an instructive developmental niche and reveal how transient tissue architecture can shape the cellular composition and function of mature organs.

## Introduction

How tissue architecture influences cell fate remains a fundamental question in organogenesis. Increasing evidence suggests that morphogenesis and fate are reciprocally coupled, with mechanical forces feeding into gene regulatory networks that influence cell identity.^1,2^ The developing pancreas provides a powerful model to study this relationship because perturbations in epithelial remodeling profoundly alter lineage allocation.^3,4^ During pancreas formation, a transient, stratified epithelium remodels into a branched organ that generates endocrine, acinar, and ductal lineages.^5^ This transition is driven by *de novo* lumen formation, whereby nascent microlumens coalesce into an epithelial plexus.^6^ Because endocrine cell production during development establishes the endocrine reserve of the adult pancreas,^7^ understanding how epithelial remodeling influences lineage allocation has important implications for lifelong organ function. Whether this transient architectural state merely accompanies differentiation or actively instructs lineage allocation is unknown.

In epithelial model systems, lumen formation begins at an Apical Membrane Initiation Site (AMIS) at the interface of two adjacent cells.^8–11^ At this site, polarity complexes and polarized membrane trafficking cooperate to establish the nascent apical domain and initiate lumen formation. These events are accompanied by junctional remodeling that stabilizes the emerging apical surface. Although AMIS formation and trafficking pathways have been characterized in *in vitro* epithelial cyst models, these processes *in vivo* during organogenesis have received less attention.^3,11–13^

Epithelial morphogenesis is increasingly recognized as being coupled to mechanotransduction pathways that sense and respond to tissue architecture. Studies from 3D culture of pancreatic progenitors demonstrate that lumen formation is highly sensitive to mechanical forces, including lumen pressure.^14^ The Hippo signaling pathway has been identified as a key sensor of physical cues, linking cytoskeletal tension and cell-cell adhesion to transcriptional control of growth and differentiation.^15–19^ In addition, PI3K signaling has been shown to regulate pancreatic epithelial organization, intracellular transport, and mechanotransduction.^20,21^ These observations raise the possibility that mechanotransduction and membrane trafficking cooperate to build transient architectures that instruct lineage allocation. Notably, recent work identified NF2 as a potent tumor suppressor in pancreatic ductal adenocarcinoma (PDAC),^22^ yet how its encoded protein MERLIN normally functions in epithelial organization is unknown.

Here, we investigate the role of the upstream Hippo pathway component MERLIN in coordinating epithelial remodeling and lineage specification during pancreatic development. We show that MERLIN localizes to nascent lumens and is required for proper lumen formation and epithelial remodeling. Pancreas-specific deletion of *Nf2* disrupts lumen morphogenesis, prevents epithelial destratification, and alters lineage allocation. Single-cell transcriptomic analysis reveals upregulation of both Hippo and PI3K signaling pathways in mutant epithelia, and genetic deletion of Yap1/Taz or pharmacological inhibition of PI3K partially rescues these defects. Together, our findings identify a morphogenetic stage in which MERLIN-dependent epithelial remodeling establishes a transient 3D architecture that determines lineage allocation and adult endocrine mass.

## Results

### Loss of Merlin impairs pancreatic growth and function.

**Fig.1.**
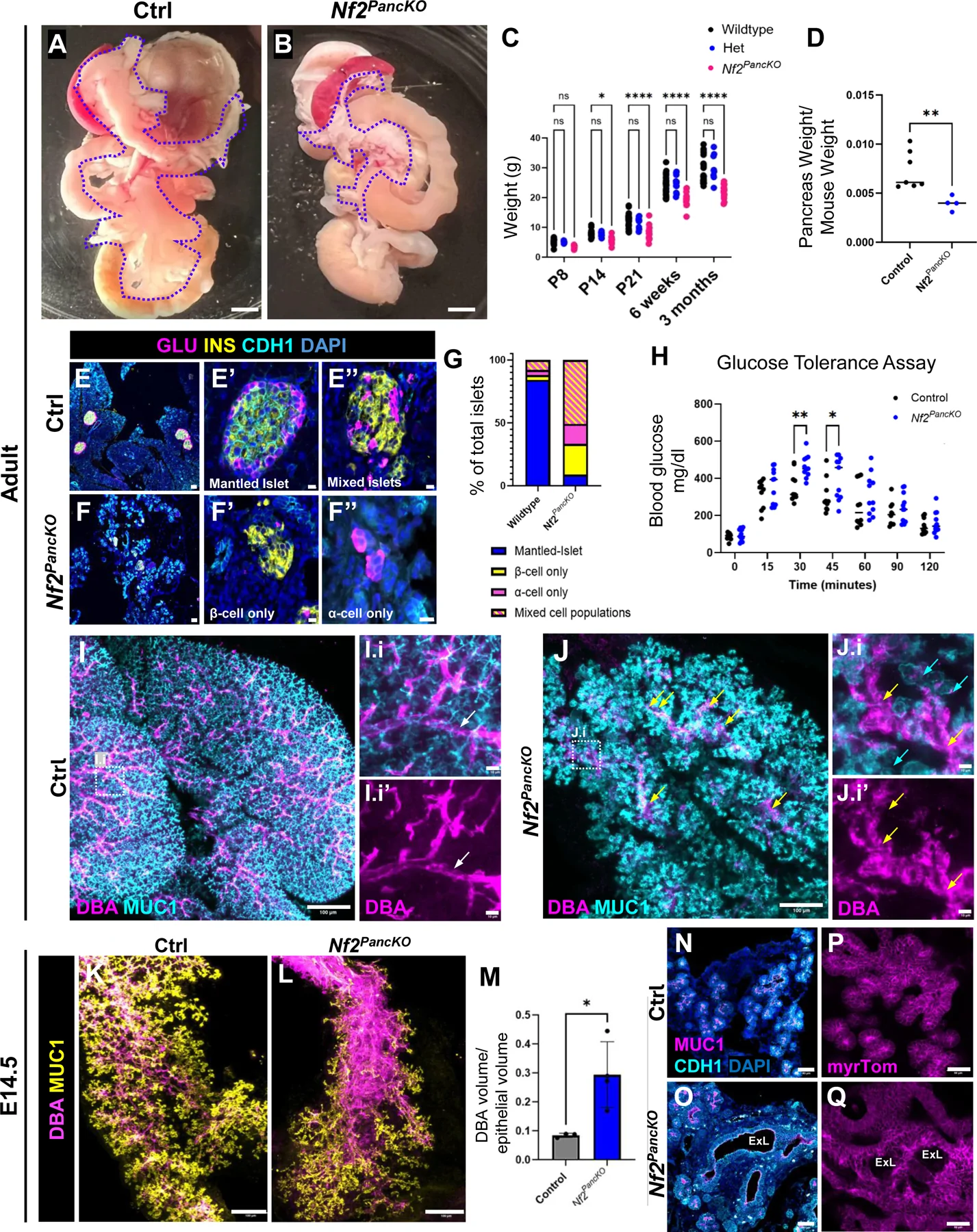
Loss of Merlin impairs pancreas growth, differentiation, and function. **A, B)** Gross morphology of digestive tract from ~16 weeks old control (A) and *Nf2*^*PancKO*^ (B) mice. Pancreata are outlined with a purple dashed line. **C)** Body weights of control, heterozygous, and *Nf2*^*PancKO*^ mice from P8 to adulthood. Data analyzed by 2-way ANOVA followed by Dunnett’s multiple comparisons test (ns indicates p>0.05, * indicates p <0.05, **** indicates p < 0.0001). **D)** Pancreatic weights normalized to body weight in 16–18 week-old mice. N= 7 ctrl (4 males, 3 females), and 4 mutants (3 males, 1 female). P=0.003 by Welch’s t-test. Immunofluorescence (IF) against GLU, INS, and CDH1 on adult control **(E)** and mutant **(F)** pancreata. **E’)** Representative control islet displaying the typical mantle of glucagon-positive α-cells surrounding inner insulin-positive β-cells. **F’-F’”)** Representative dysmorphic *Nf2*^*PancKO*^ islets. **G)** Quantification of islet morphology in control and *Nf2*^*PancKO*^. (n=17 control, 17 mutant islets evaluated from n=3 ctrl, 3 mutant animals between 16–18 weeks). **H)** Intraperitoneal glucose tolerance test in 15–18-week-old control and *Nf2*^*PancKO*^ mice. N= 10 ctrl (4 male, 6 female), 11 *Nf2*^*PancKO*^ (5 male, 6 female). Analyzed by 2-way ANOVA, with Tukey’s multiple comparison test, p= 0.0022 at 30 minutes, and 0.0333 at 45 minutes. Other time points were not significantly different. **(I-J.i’)** WMIF of DBA and MUC1 of a single branch from the splenic lobe of control (white arrow, large duct) **(I-I.i’)** and mutant **(J-J.i’)** adult pancreas. Yellow arrows, DBA^+^ lesions; cyan arrows, MUC1^+^ dilated lumens. Whole mount immunofluorescence (WMIF) of control **(K)** and *Nf2*^*PancKO*^ **(L)** E14.5 pancreata, stained for MUC1^+^ and DBA. **M)** Quantification of ductal volume/epithelial volume (N = 3 ctrl, 4 mutant pancreata. p=0.03 by Welch’s t-test). Representative images in K,L are shown from E14.5 pancreata; quantification includes pooled E13.5–E14.5 littermate pancreata. Error bars indicate standard deviation (std). **N-Q)** Section IF of control (**N, P)** and mutant (**O, Q)** pancreata at E14.5 (100μm vibratome sections). Scale bars: Panels A and B 100 mm; E and F 130μm; E’-F” 25μm; I, J, K and L 100μm; I.i, I.i’, J.i, and J.i’ 10μm, and N-Q 50μm.

To investigate the role of MERLIN in pancreas formation, we generated pancreas-specific Nf2 knockout mice (*Nf2*^*f/f*^*;Pdx1*^*Cre*^, or *Nf2*^*PancKO*^). Although *Nf2*^*PancKO*^ mice survived to adulthood (**fig. S1A**), they exhibited marked pancreatic hypoplasia (Fig. 1A, B and **fig. S1B**). *Nf2*^*PancKO*^ mice also displayed reduced body weights beginning at P14; however, pancreatic mass was disproportionately reduced relative to body weight (Fig. 1C, D). Pancreatic dysplasia was evident by embryonic day (E)14.5, but not E11.5, when epithelial organization and progenitor cell morphology appeared grossly normal (**fig. S1C-K.i**). Smaller pancreas size was not associated with detectable changes in proliferation (phospho histone H3, pHH3) or apoptosis (Cleaved Caspase 3, CC3), at either E14.5 or P1 (**fig. S1L-W**). Rare apoptotic cells were observed adjacent to cystic lumens in mutant pancreata (**fig. S1U, U’**).

Because adult *Nf2*^*PancKO*^ mice were smaller, we asked whether pancreas endocrine mass and function were impaired. We assessed endocrine cells by immunofluorescence (IF) for endocrine hormone markers. Control islets displayed the expected organization of peripheral glucagon-positive alpha (α) cells surrounding a core of insulin-producing beta (β) cells^23^ (Fig. 1E, E’), although occasional islets with mixed populations were also observed (Fig. 1E”). By contrast, *Nf2*^*PancKO*^ islets were dysmorphic, smaller, and often displayed single hormone positivity, or intermingled α- and β-cells (Fig. 1F–F”), with fewer β-cells (Fig. 1G, **fig. S2A-C**). Somatostatin-producing delta (δ) were unchanged, and pancreatic polypeptide-producing gamma (γ) cells were only modestly reduced (**fig. S2D-I**). Consistent with these islet defects, *Nf2*^*PancKO*^ mice showed impaired glucose tolerance (Fig. 1H), with a more pronounced defect in females than in males (**fig. S2J, K**).

### Nf2^PancKO^ pancreata display defects in embryonic epithelial architecture.

Because endocrine progenitors arise from a proto-differentiated ductal epithelium,^24^ we examined ductal morphogenesis. Whole Mount ImmunoFluorescent microscopy (WMIF) for the ductal marker Dolichos Bioflorus Agglutinin (DBA) revealed a highly branched ductal network in controls, with large central DBA^+^ ducts connecting to fine, peripheral Mucin-1^+^ (MUC1^+^) lumens (Fig. 1I–I.i’). By contrast, *Nf2*^*PancKO*^ pancreata displayed diffuse DBA and dilation of terminal MUC1^+^ ducts (Fig. 1J–J.i’). These defects were evident prior to birth at E14.5 (Fig. 1K–M), where mutant ducts exhibited markedly expanded lumens (ExL; Fig. 1N–Q).

### Loss of Merlin impairs epithelial destratification.

During pancreas formation, a transiently stratified bud epithelium progressively resolves as the branched ductal network takes shape (**fig. S3A**).^6,25,26^ We previously showed that destratification is driven by *de novo* microlumen formation and lumen extension.^6,27^ We found that MERLIN was expressed at the earliest stages of MUC1^+^ pancreatic lumen formation (**fig. S3B-B.i”**). Of note, Pdx1^Cre^ did not reduce Merlin expression at this early stage (**fig. S3C-C.i”**), but did so by E11.5 (**fig. S3D-E.i”**)^28,29^ after initial lumens have already formed.

**Fig. 2.**
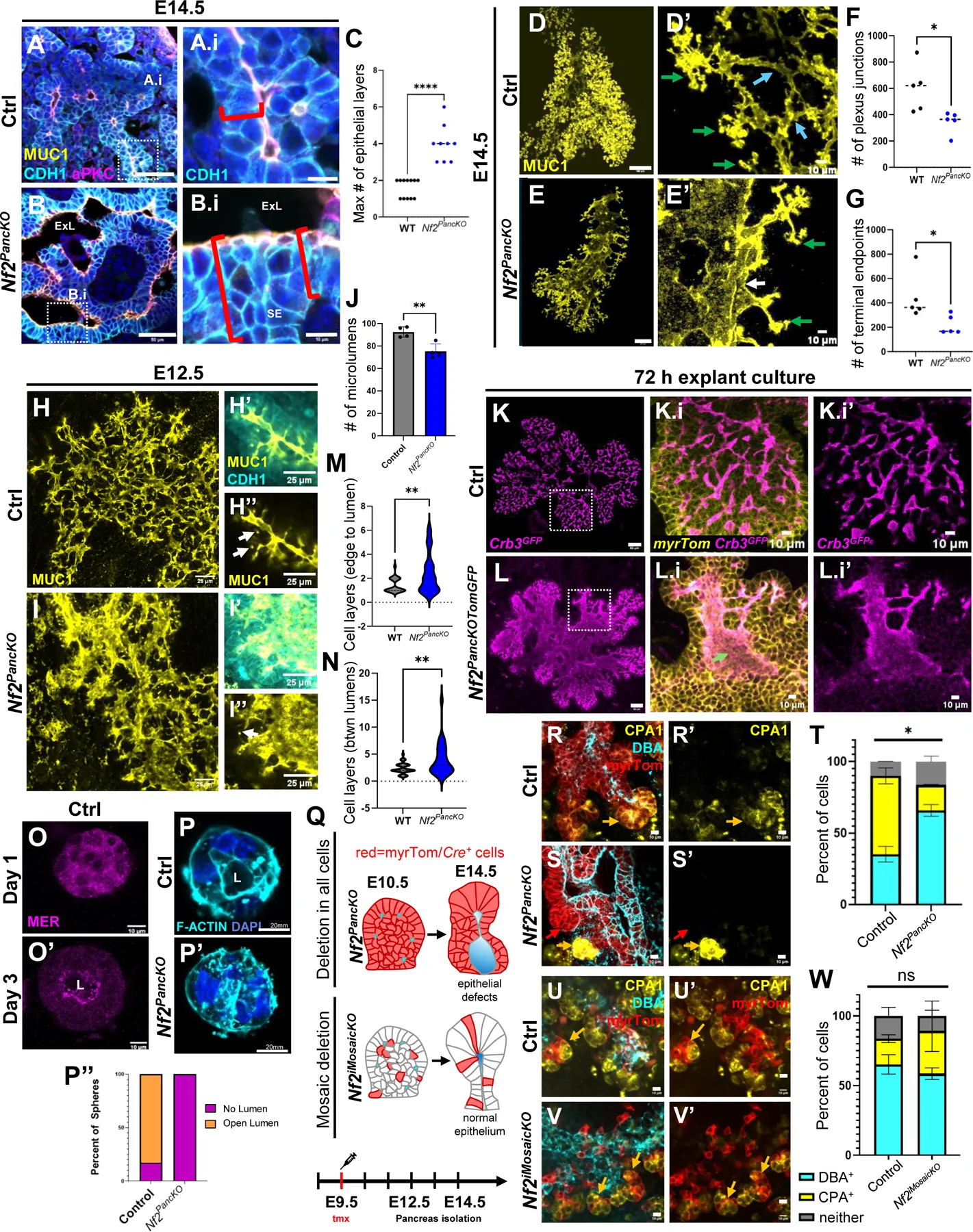
Pancreatic lumen formation and epithelial remodeling require MERLIN. **A-B.i)** Representative 10 μm sections of control (A) and mutant (B) pancreata. Red brackets mark epithelial layers between apical surfaces and epithelial edge. **C)** Quantification of maximum epithelial layers per field of view (FOV) (n=4 control, 3 *Nf2*^*PancKO*^ mutants; ****p < 0.0001, unpaired t-test). **D, E)** WMIF Max Intensity Projections (MIPs) of E14.5 control (D) and *Nf2*^*PancKO*^ (E) luminal networks. **D′, E′)** High magnification substacks highlight plexus junctions (blue arrows) and terminal endpoints (green arrows). **F, G)** Quantification of lumen junctions and terminal endpoints per pancreas (p = 0.0207, 0.0382, unpaired t-test). Each dot represents an individual animal. **H, I)** WMIF at E12.5. **H′, I″)** High magnification views showing *de novo* microlumens (white arrows). **J)** Quantification of microlumen number per pancreas (p = 0.0071, Welch’s t-test, each dot represents an individual animal). **K, L)** MIPs of explants after 72 h culture. Boxes indicate regions shown in optical sections in **K.i–L.i′**. Green arrow indicates enlarged lumen. **M, N**) Quantification of epithelial layers from edge to lumen (M) and between lumens (N) (40 control, 30 mutant measurements from 4 control, 3 mutant explants; p = 0.0033, 0.0061, Welch’s t-test). **O)** WMIF shows MERLIN localization in wildtype spheres at Day 1 and Day 3, becoming strongly apical by Day 3. L, lumen. **P)** Pancreatospheres generated from E12.5 control and mutant tissue (96 h culture), stained for F-actin and DAPI. Mutant spheres fail to form central lumens (n = 23 control spheres/3 embryos; 20 mutant/2 embryos). **Q)** Experimental strategy for lineage tracing and mosaic deletion. Tamoxifen (tmx) induction at E9.5 yields ~10% labeled epithelial cells by E11.5–E14.5, compared to widespread labeling in *Nf2*^*PancKO*^. **R, S)** Vibratome sections stained for DBA, CPA1, and myrTom(RFP) show increased ductal (DBA^+^) and reduced acinar (CPA1^+^) fate in *Nf2*^*PancKO*^ (S, S’). Orange arrows, some myrTom^+^ cells show expression of CPA1; red arrows, most myrTom^+^ cells show no CPA1 stain; red arrows (N=2344 control, 2851 mutant cells from 2 embryos/genotype; two-way ANOVA followed by Tukey’s test; P values as follows: DBA+ Control vs *Nf2*^*PancKO*^, <0.0001; CPA + Control vs *Nf2*^*PancKO*^ <0.0001, Neither Control vs *Nf2*^*PancKO*^, 0.3463.**U-V’)** Optical sections from vibratome sections of control (U, U’) and *Nf2*^*iMosaicKO*^ (V, V’) pancreata stained for DBA, CPA1 and (RFP). **W)** Quantification of lineage allocation following mosaic Nf2 deletion (N=715 control, 460 mutant cells evaluated from n=5, 4 embryo respectively. P=0.88 by two-way Anova). For T,W, error bars indicate standard deviation. Scale bars: Panels A, B, K and L 50μm;D,E 50μm, A.i, B.i, D’, E’, K.i-L.i’, O, O’, R-S’ and U-V’ 10μm; H-I” 25μm; P, P’ 20μm.

By E14.5, control pancreata consist largely of polarized epithelial monolayers surrounding narrow lumens.^6^ We found that, by contrast, *Nf2*^*PancKO*^ pancreata retained extensive stratified epithelium (SE) and exhibited expanded lumens (Fig. 2A–C, **and movies S1 and S2**). Light sheet imaging of WMIF confirmed a significant increase in epithelial cell layers around mutant lumens in 3D (**movies S3 and S4**).

### Pancreatic plexus formation is dependent on Merlin.

Endocrine progenitors arise and exist solely within the pancreatic plexus, and perturbation of this epithelial structure alters pancreatic differentiation.^4,6,19^ This plexus provides a unique niche that incubates endocrine progenitors.^27,30^ 3D WMIF for MUC1 at E14.5 revealed marked plexus defects in *Nf2*^*PancKO*^ pancreata, including fewer lumenal junctions and tips (Fig. 2D–G, **movies S5-S6**). These defects were evident by E12.5, when mutant pancreata formed significantly fewer microlumens (Fig. 2H–J).

To visualize epithelial dynamics, we performed live imaging of explanted pancreata.^4,31,32^ Explants expressed myristoylated-Tomato (*myr*^*Tom*^) to outline cell membranes, and Crumbs3-GFP (*Crb3*^*GFP*^) to mark apical lumen surfaces.^33^ After 72 hours (hrs) of culture, *Nf2*^*PancKO*^*;myr*^*Tom*^*;Crb3*^*GFP*^ (termed here *Nf2*^*PancKOTomGFP*^) explants retained stratified epithelium and displayed dysmorphic lumens (Fig. 2K–N). Although mutant and control explants grew at similar rates, mutant epithelium failed to destratify and showed branching defects by 3 days of culture (**fig. S4A-G**). Epithelial clefts, which presage pancreatic branches,^34^ formed at similar rates but were reduced by ~40% in mutant explants at 48 and 72 hrs (**fig. S4H**). Overall, these experiments show that loss of Merlin results in aberrant morphogenesis of the pancreatic epithelium, and to impaired isletogenesis (**fig. S5**).

### Merlin is required for epithelial lumen formation.

**Fig 3:**
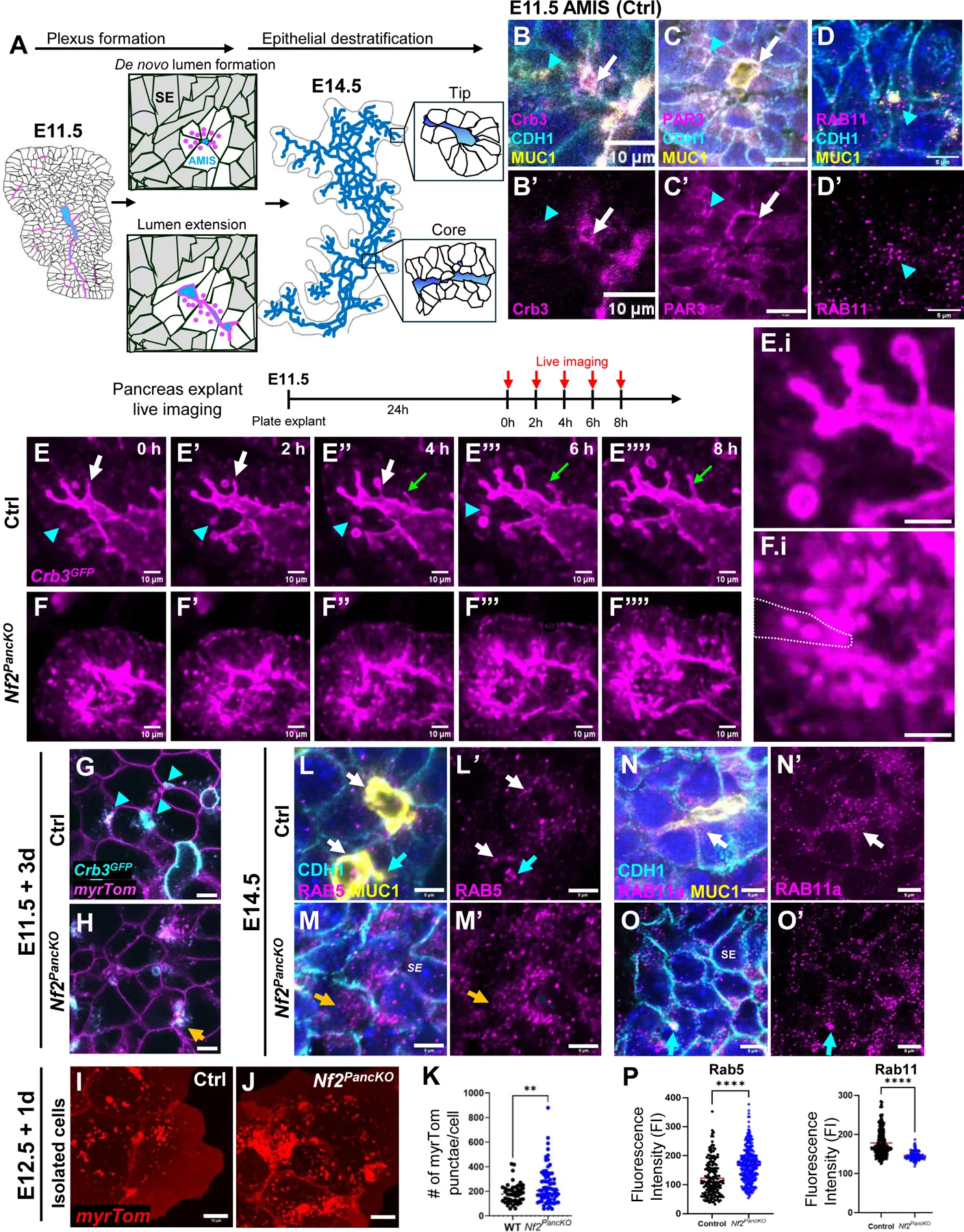
MERLIN coordinates polarized vesicular trafficking at the AMIS during pancreas lumenogenesis. **A)** Model of pancreatic lumenogenesis. At E11.5, the pancreatic epithelium is transiently stratified and undergoes *de novo* lumen formation and lumen extension via polarized vesicle trafficking. Progressive lumen growth and fusion drives destratification into an epithelial monolayer by E14.5. **B-D’)** IF showing localization of canonical AMIS markers Crb3, Par3, and Rab11 at nascent pancreatic lumens. **E-F””)** Live imaging (0–8 h; MIPs; Videos 3–4) of control (E) and *Nf2*^*PancKO*^ (F) pancreata. Control explants exhibit *de novo* microlumen formation (cyan arrowheads), lumen fusion (white arrows), and lumen extension (green arrows), whereas mutants exhibit lumen disruption and defective Crb3 trafficking. **E.i, F.i)** Higher magnification views showing Crb3 localization. **E)** In control explants, Crb3 is tightly restricted to the apical membrane, while **F)** mutants accumulate intracellular Crb3 aggregates. White dashed line indicates a single cell boundary extrapolated from myrTom labeling (see Supplementary videos). Representative images from n=3 controls and 6 mutants evaluated. Representative images from control **(G)** and mutant **(H)** explants. Mutant explants display a disorganized luminal network and intracellular Crb3. Representative images from n = at least 3 pancreata per genotype. **I, J)** Primary epithelial cells isolated from E12.5 control (I) and mutant (J) pancreata, cultured for 24 h. **K)** Quantification of intracellular myrTom+ structures. Mutant cells accumulate significantly more intracellular vesicles than controls (n = 48 control cells from 2 pancreata, and 73 mutant cells from 3 embryos. P=0.0044 by Welch’s t-test). **L-M’)** IF for RAB5 in E14.5 control and mutant pancreata. RAB5 is localized along the apical membrane in control pancreata (white arrows, L, L’). In mutant stratified epithelium (SE) regions (M, M’), RAB5 is accumulates at nascent lumens (cyan arrows) and in the cytoplasm (orange arrow). **N-O’)** In mutant SE at E14.5, RAB11 levels are decreased (representative images from n=2 embryos per genotype). **P)** Quantification of RAB5 and RAB11 fluorescence intensity/cell (N > 100 cells analyzed per genotype, p <0.0001 for both RAB5 and RAB11 by Welch’s t-test. Red line indicates mean). Scale bars: Panels B-F.i’ and I-J 10μm; G,H L-O’ 5μm.

Our previous studies showed that E9.75-E12.5 pancreatic epithelium continuously generates *de novo* lumens that interconnect to form the ductal plexus (Fig. 3A).^4,6^ Because *Pdx1*^*Cre*^ deletes *Nf2* around E11.5 after initial lumens have already formed, we used a pancreatosphere assay to test the direct requirement for MERLIN in *de novo* lumen formation.^4,35^ This assay involves isolation of E12.5 pancreas control or mutant cells (with already deleted *Nf2*) and assessment of *de novo* lumen formation. In pancreatospheres, MERLIN became enriched at nascent apical lumen membranes by 3 days of culture (Fig. 2O, O’). Control spheres formed single central lumens by 96 hrs, while mutant spheres did not (Fig. 2P–P”). These data show that Merlin is directly required for *de novo* lumen formation, suggesting that failure of destratification evident in *Nf2*^*PancKO*^ likely results from impaired lumenogenesis following Cre-mediated deletion. Moreover, they suggest that defective lumenogenesis underlies failure of epithelial remodeling in mutants.

### Disruption of epithelial architecture alters cell fate non-cell autonomously.

Our previous studies showed that pancreatic cell fate depends on epithelial morphogenesis.^3,4,36^ To establish if Merlin regulates lineage allocation, we lineage-traced control and *Nf2*^*PancKO*^ cells from the onset of Pdx1^**Cre**^ expression (~E8.75) through E14.5 (Fig. 2Q). In controls, 38% of labeled cells adopted a ductal (DBA^**+**^) fate, whereas 53% became acinar (CPA1^**+**^) (Fig. 2R, R’, T). By contrast, in *Nf2*^*PancKO*^ pancreata, 71% of labeled cells became ductal and only 19% became acinar, indicating a marked shift towards ductal identity (Fig. 2S, S’, T).

To distinguish cell-autonomous from tissue-level functions of MERLIN, we generated mosaic Nf2 deletions and compared them with *Nf2*^*PancKO*^ (Fig. 2Q). Mosaic deletion was induced at E9.5 in *Ptf1a*^*CreERT2*^*;Nf2*^*f/f*^*;myrTom*^*f/f*^ mice (or *Nf2*^*iMosaicKO*^) using low-dose tamoxifen (tmx) labeling of a subset of epithelial cells (Ptf1a is expressed broadly in epithelium at this stage^37^). This strategy labeled approximately 10% of epithelial cells. Importantly, lineage allocation was unchanged in *Nf2*^*iMosaicKO*^ mutants, with similar proportions of Tom^+^ cells adopting ductal or acinar fates in both control and mutant mosaics. Thus, loss of MERLIN in individual cells did not alter fate, while loss in all cells skewed lineage proportions. These data demonstrate that MERLIN regulates lineage allocation non-cell autonomously, indicating that disruption of epithelial architecture, or the cellular neighborhood, rather than loss of MERLIN in individual cells, drives the fate defects observed in *Nf2*^*PancKO*^ pancreata.

### Merlin is expressed at the pre-lumen Apical Membrane Initiation Site (AMIS).

In MDCK cells and in developing kidney, *de novo* lumen formation begins at an Apical Membrane Initiation Site (AMIS) where Crb3, Par3, and Rab11^+^ vesicles promote apical membrane biogenesis (**fig. S5.A**).^8,11,13,38^ These observations underscore the dependence of lumen formation on polarized membrane trafficking. Consistent with this, Crb3, Par3, Par6, Rab11, Merlin, and Muc1 were enriched at pancreatic AMISs and nascent lumens (Fig. 3B–D’ and **fig. S5**). These data indicate that the same AMIS machinery operates in developing pancreatic epithelium *in vivo*.

### MERLIN is required for apical targeting of Crb3.

To test whether Merlin is required for AMIS formation, we examined apical polarity proteins in control and *Nf2*^*PancKOTomGFP*^ pancreatic explants cultured *ex vivo*. Loss of MERLIN disrupted both *de novo* lumen initiation (arrowheads) and lumen extension (arrows) (Fig. 3E–F.i, and **movies S7 and S8**). Moreover, Crb3 failed to accumulate at the apical membrane and instead remained intracellular (Fig. 3G, H and **fig. S6A-F.i**). In controls, Crb3 was tightly restricted to the apical surfaces of nascent and extending lumens, whereas mutant epithelia displayed diffuse intracellular and basolateral Crb3 accumulation. When microlumens formed in mutant epithelia, they were sometimes intracellular (**movies S9 and S10**). These findings indicate MERLIN is required for apical membrane assembly during pancreas lumenogenesis.

### Merlin is required for AMIS and *de novo* lumen formation, but not for maintenance of existing apical identity.

In the persisting stratified epithelium of *Nf2*^*PancKO*^, Muc1^+^ lumens were absent (**fig. S6G-H.i’**). However, Par3 remained expressed and was scattered along the cell cortex. This suggests that Merlin is required to focus polarity components into discrete AMIS and initiate lumen formation.

Loss of Merlin produced two distinct epithelial phenotypes: 1. Perduring stratified epithelium (SE), and 2. Expanded lumens (ExL) (Fig. 2A–C and **fig. S7**). These phenotypes likely reflect the timing of *Nf2* deletion by Pdx1^Cre^ (see fig. S4). Epithelial cells that lose MERLIN before AMIS establishment fail to initiate *de novo* lumen formation altogether, whereas microlumens that form before Merlin depletion expand. In expanded mutant lumens, MUC1^+^ apical cells retained normal localization of aPKC and ZO-1, whereas these markers were absent from Muc1- stratified cells (**fig. S8A-D.i”, E-H.i”**). We next evaluated cell polarity using Golgi positioning. In control epithelia, the Golgi (anti-Golgi matrix protein 130, GM130) localized beneath the apical membrane (**fig. S8I-I.i”**). Similarly, mutant cells that had an apical surface also displayed normal subapical Golgi (**fig. S8J-J.i”**), similar to ZO1 and aPKC.

### Loss of MERLIN uncouples intracellular polarity from apical domain assembly.

Despite disrupted Par3 localization, stratified *Nf2*^*PancKO*^ cells retained polarized Golgi positioning (**fig. S8I-L.ii**). In control epithelia, the Golgi localized to beneath AMIS-associated Par3, while in mutant stratified regions Golgi and Par3 positioning became uncoupled, with individual cells displaying multiple Par3 foci. By contrast, the apical markers ZO-1 and aPKC were absent from mutant stratified epithelium (**fig. S8E-L.i’**). These data indicate that loss of MERLIN disrupts assembly of a coherent apical domain without abolishing intracellular polarity (much like loss of Cdc42),^25^ leaving a polarized Golgi axis intact.

### MERLIN coordinates productive apical membrane trafficking.

Because Crb3 transport was disrupted in *Nf2*^*PancKO*^ epithelia (**movies S11 and S12**), we quantified intracellular vesicles in isolated pancreatic epithelial cells. E12.5 control and *Nf2*^*PancKOTomGFP*^ pancreatic buds were dissociated and cultured on glass coverslips for vesicle analysis (Fig. 3I–K). Mutant cells exhibited a ~33% increase in intracellular Tom^+^ vesicles, indicating accumulation of intracellular membrane compartments.

Crb3 is an early apical determinant required for lumen formation and is trafficked to nascent apical membranes during epithelial polarization.^38^ It is one of the first components recruited to forming lumens in Madin Darby Canine Kidney (MDCK) epithelial cysts.^8,39^ Live imaging revealed elevated vesicle trafficking near nascent lumens compared with established lumens, consistent with a role in lumen assembly (**movies S13 and S14**).

Because Crb3 is known to be trafficked to the AMIS via Rab11^+^ vesicles, we examined endosomes by IF in control and *Nf2*^*PancKO*^ pancreas. Loss of MERLIN resulted in marked increase in Rab5^+^ endosomes and a concomitant reduction in Rab11^+^ recycling endosomes at E14.5, both in intact pancreata and isolated epithelial cells (Fig. 3L–P, **fig. S9.**). Moreover, both Rab5^+^ and Rab11^+^ vesicles lost their normal subapical enrichment and were dispersed throughout the cytoplasm. This redistribution was confirmed by altered apical:basal fluorescence intensity (FI) ratios in lumen-lining (**fig. S9H**). Similarly, the trafficking components Clathrin and Cdc42, which normally localize to nascent apical domains, became mislocalized in stratified regions of *Nf2*^*PancKO*^ by E14.5 (**fig. S9I-J.i’**). These defects were more pronounced in central regions of the epithelium in mutants (**fig. S10**). Together, these data support a model wherein Merlin promotes productive apical membrane trafficking by coordinating endocytic and recycling pathways during lumen initiation.

### Loss of MERLIN alters epithelial lineage allocation.

To define the molecular consequences of *Nf2* loss, we performed single-nucleus RNA sequencing (snucRNAseq) of control and mutant pancreata. We first profiled control E14.5 pancreata (**fig. S11. A-B**), when mutant defects are already evident (see Fig. 1G–M). UMAP analysis of control and mutant E14.5 pancreata (3–4 per genotype) revealed expansion of ductal cells, loss of acinar cells, and a modest increase in mature endocrine cells (**fig. S11. A-C**).

Compared with controls, *Nf2*^*PancKO*^ pancreata showed increased progenitor gene signatures (**fig. S12**). Because pancreatic multipotent progenitors (MPs) are normally largely depleted by E14.5,^40^ they were not detected in our control dataset. To account for this developmental shift, we bioinformatically integrated data from both E11.5 and E14.5 control datasets for comparison (**fig. S13**). Pseudotime analysis revealed two principal paths to endocrine cell fate, likely reflecting the first- and secondary-wave endocrine differentiation (**fig. S13**). These analyses identified seven distinct epithelial cell populations at E14.5, including multipotent progenitors, acinar (Aci), ductal (Duc), endocrine progenitor (EP), and endocrine cells (End).

**Fig 4.**
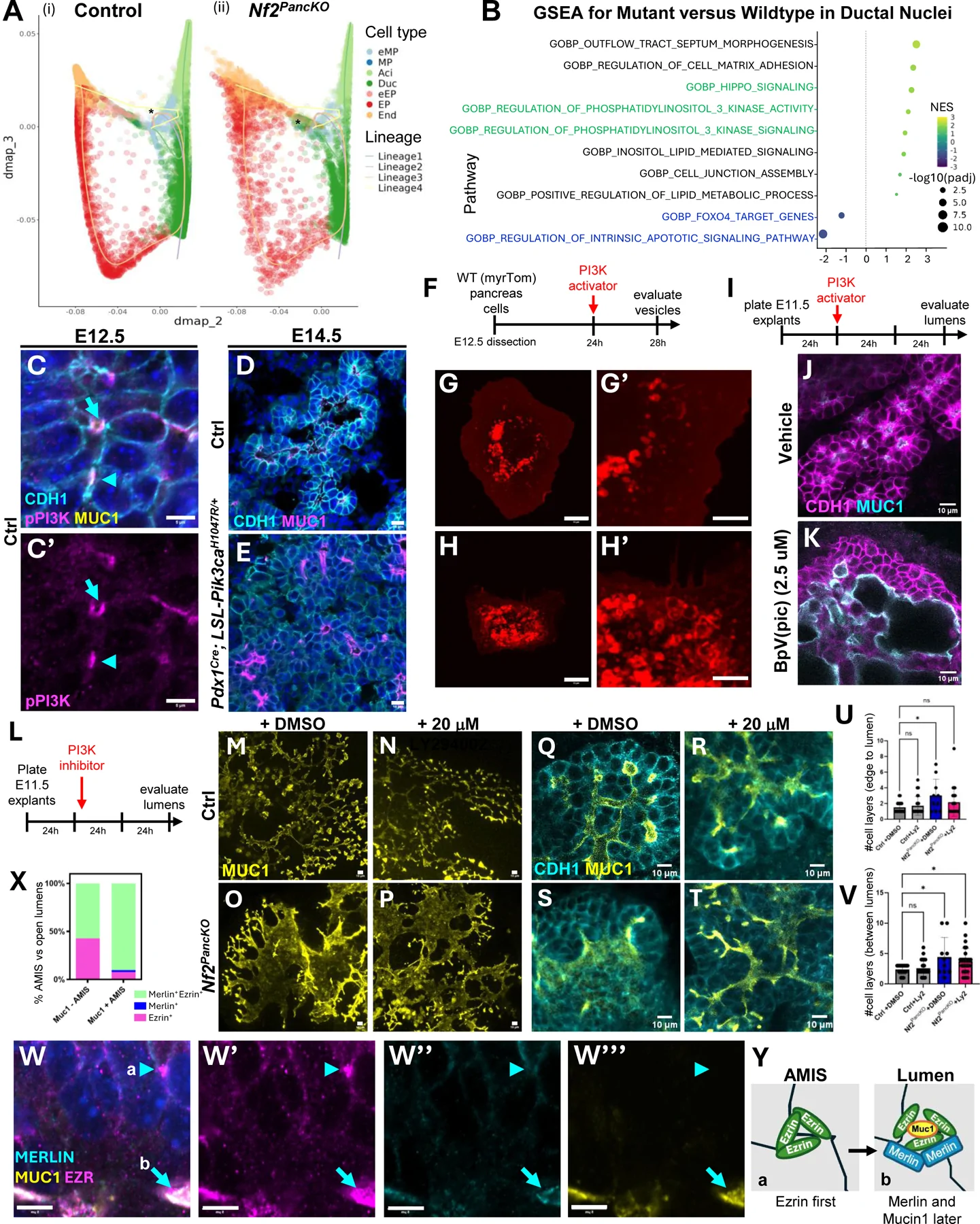
MERLIN restrains Hippo and PI3K signaling to coordinate polarized trafficking and epithelial morphogenesis. **A)** Lineage trajectory analysis of control and *Nf2*^*PancKO*^ single-nucleus RNA-seq data (* indicates the inferred trajectory root). **B)** Gene set enrichment analysis (GSEA) of ductal nuclei reveals activation of Hippo and PI3K pathways (green) and repression of FOXO and apoptosis programs (blue) following loss of MERLIN. **C)** Phosphorylated PI3K (p85/p55) localizes to the apical membrane of nascent lumens (AMIS, cyan arrowhead; Opening lumen, cyan arrow). **D, E)** Representative E14.5 control (D) and Pik3ca-overexpression (*Pdx1*^*Cre*^*; LSL-Pik3caH1047R*) (E) pancreata (n=3 embryos/ genotype). Constitutive PI3K activation phenocopies epithelial stratification defects seen in mutants. **F)** Experimental design for isolation of E12.5 myrTom+ pancreatic cells and treatment with vehicle or 2.5 μM of the PI3K activator BpV(pic). **G, H)** Low magnification and **(G’, H’)** high magnification views of intracellular Tom+ vesicles in control and BpV(pic) treated cells, showing increased and enlarged vesicles in the treated cells. **I)** Experimental design for E11.5 pancreatic explants cultured for 24 h and treated with vehicle or 2.5 μM BpV(pic) (PI3K activator) for an additional 48 h. **J, K)** PI3K activation blocks epithelial de-stratification and induces dysmorphic lumens. **L)** Experimental design for LY294002 rescue experiments. Explants treated with the PI3K inhibitor LY294002 at 24 h and analyzed at 72 h. **M-P)** MIPs of MUC1 immunofluorescence (IF) in control (M, N) and mutant (O, P) explants treated with DMSO or 20 μM LY294002. **Q-T)** High-magnification optical sections stained for CDH1 and MUC1. **U, V)** Quantification of epithelial layering from epithelial edge to nearest lumen (U) and between adjacent lumens (V). PI3K inhibition partially rescues the stratification phenotype in mutants. U) Data analyzed by one-way ANOVA with Dunnett’s test (P-value for control-DMSO vs control-Ly2 = 0.93, control-DMSO vs *Nf2*^*PancKO*^-DMSO = 0.02, control-DMSO vs *Nf2*^*PancKO*^-Ly2 = 0.29). V) Data analyzed by ANOVA followed by Dunnet’s test. P-value for control-DMSO vs control-Ly2 = 0.95, control-DMSO vs *Nf2*^*PancKO*^-DMSO = 0.01, control-DMSO vs *Nf2*^*PancKO*^-Ly2 = 0.03). For both U and V: n = 20 measurements from 2 control+DMSO explants, 30 from 3 control+Ly2, 10 from 1 *Nf2*^*PancKO*^+DMSO, and 25 from 3 Nf2PancKO+Ly2 explants. Error bars indicate standard deviation. **W-W”)** IF showing EZR and MERLIN localization at AMIS (cyan arrowhead) and at nascent lumens (cyan arrow) in E11.5 pancreata. **X)** Quantification of MERLIN and EZR at early, MUC1-negative AMIS and more developed MUC1-positive AMIS at E11.5 (66 total AMIS evaluated, from n=3 pancreata). **Y)** Schematic depicting AMIS maturation, with EZR arriving earlier than MERLIN. Scale bars: Panels C-C’ and T-T” 5μm, D, E, G, H, J-Q 10μm.

To examine differentiation dynamics, we performed lineage trajectory analysis (Fig. 4A). Diffusion maps resolved four main lineages: acinar, ductal, and two different endocrine trajectories. One endocrine trajectory likely corresponds to first-wave endocrine cells (the primarily glucagon^+^ cells that arise as clusters in the embryonic pancreatic bud between E10.0-E12.5), while the other represents secondary-wave endocrine cells (the singly delaminating endocrine cells that give rise to pre- and perinatal endocrine cells).^41^

In *Nf2*^*PancKO*^ pancreata, pseudotime analysis suggested a modest increase in MPs and impaired maturation along both endocrine trajectories (**fig. S15A-B’** and **Supplementary fig. 2E**). Quantification of endocrine cells at E14.5 identified by WMIF revealed a transient increase in mass that was abolished by P1 (**fig. S15C-K**). By IF performed on tissue sections, however, endocrine progenitors (EP) (Neurog3^+^) and the β-to-α-cell ratio were relatively unchanged at midgestation, although there was a modest reduction of the latter (**fig. S15L-Q**). Collectively, these findings indicate subtle embryonic endocrine defects in *Nf2*^*PancKO*^ pancreata, with the major deficit emerging postnatally.

By contrast, acinar loss was already pronounced in E14.5 *Nf2*^*PancKO*^ pancreata. Expression of acinar genes, including *Amy2a1* and *Cpa1*, was markedly reduced (**fig. S16A**). Consistent with this, acinar mass was decreased ~3 fold by IF staining for carboxypeptidase A1 (CPA1) and amylase (AMY, **fig. S16B-G**). Acinar defects persisted after birth, with co-expression of acinar and ductal markers at P1 (**fig. S16H-I)**. Key acinar transcription factors, including *Ptf1a*, *Foxa2* and *Gata4*, were also significantly downregulated at E14.5 (**fig. S16J**).

### MERLIN defines a developmental window for pancreatic cell fate determination.

To determine whether Merlin is required after pancreas morphogenesis, we deleted *Nf2* following epithelial remodeling and lineage allocation, at mid-gestation. Nf2 was deleted in acinar cells at E14.5, using *Ptf1a*^*CreERT2*^*;myrTom*^*ff*^*;Nf2*^*ff*^ (*Nf2*^*iAcinarKO*^) or *myrTom*^*ff*^*;Nf2*^*ff*^ (*Cre-*negative control). Despite efficient recombination (myrTom^+^), most acinar cells exhibited no detectable acinar or ductal defects at E17.5 (**fig. S17A-D.i”**). Likewise, deletion of *Nf2* in differentiated β-cells (*Ins*^*CreERT2*^*;myrTom*^*ff*^*;Nf2*^*ff*^ pups induced at P2 and P3, *Nf2*^*iInsKO*^) did not affect insulin expression or islet architecture (**fig. S17E-F.i”**). The evidence supports a model whereby Merlin is critically required during a restricted developmental window but is dispensable for maintenance of differentiated pancreatic cell fates.

### Hippo activation incompletely explains defective *Nf2*^*PancKO*^ morphogenesis.

Gene Set Enrichment Analysis (GSEA) confirmed expected activation of Hippo pathway targets following loss of Merlin (Fig. 4B). Canonical Yap1/Taz targets, including *Cyr61(Ccn1)*, *Ctgf(Ccn2)*, and *Ankrd1*, were significantly upregulated (**fig. S18A, D**). Increased expression was observed in both acinar and ductal populations by snucRNAseq. This suggests that pancreatic MERLIN suppresses Yap1/Taz signaling in the developing pancreas. However, combined heterozygous loss of Yap1 and Taz only partially rescued the lumen defects of *Nf2*^*PancKO*^ pancreata (*Nf2*^*PancKO*^*;Yap1*^*f/+*^*;Taz*^*f/+*^*;Pdx1*^*Cre*^), (**fig. S19** and **movies S15-S18**), suggesting that additional pathways contribute to the morphogenetic defects caused by MERLIN loss.

### Merlin restrains PI3K-driven trafficking programs.

Transcriptional profiling further revealed activation of phosphoinositide 3-kinase (PI3K) and membrane trafficking pathways following loss of *Nf2* (Fig. 4B and **fig. S18B-C’, E-G**). Consistent with increased PI3K activity, FoxO transcription factors (*Foxo1* and *Foxo3*) and apoptosis-associated gene programs were significantly downregulated. These findings also align with previous studies identifying Merlin as a negative regulator of PI3K signaling in HEK293 cells.^42,43^ Genes encoding vesicle trafficking and endosomal transport, including Rab5, Rab8 and Exoc1, were also upregulated **(fig. S20)**.

### PI3K activation phenocopies *Nf2* loss.

We next tested whether PI3K dysregulation contributes to the lumenogenesis defects observed in *Nf2*^*PancKO*^ pancreata. We found PI3K was enriched at nascent lumens during early pancreas morphogenesis, at E12.5 (Fig. 4C, C’). To assess the consequences of elevated PI3K signaling, we genetically activated PI3K during pancreas lumen and plexus formation. Expression of constitutively activated Pik3ca (*LSL-Pik3caH1047R*)^44^ using the *Pdx1*^*Cre*^ driver caused persistent epithelial stratification and expansion of the ductal compartment, closely phenocopying *Nf2*^*PancKO*^ pancreata (Fig. 4D, E and **fig. S21**).

We next tested PI3K function pharmacologically in pancreatic isolated cells and explants. Treatment of disaggregated cells from control *myr*^*Tom*^ pancreata with the PI3K activator BpV(pic) resulted in increased intracellular trafficking, with enlarged vesicles (Fig. 4F–H’). Similarly, treatment of E11.5 explanted pancreata resulted in dilated lumens, reduced plexus complexity, and perduring stratified epithelium, recapitulating the major architectural defects associated with loss of Merlin (Fig. 4I–K). Explants containing the *Crb3*^*GFP*^ reporter and treated with BpV(pic) revealed apical membrane disorganization and enlarged intracellular Crb3^+^ vesicles (**fig. S22**). Conversely, treatment of *Nf2*^*PancKO*^ explants with the PI3K inhibitor LY294002 partially rescued epithelial morphogenesis (Fig. 4L–V). Inhibitor-treated *Nf2*^*PancKO*^ pancreata exhibited increased plexus complexity relative to vehicle-treated mutants. Together, these findings identify PI3K signaling as a key regulator of pancreatic epithelial morphogenesis and indicate that MERLIN promotes lumen formation and plexus remodeling, in part through suppression of PI3K activity.

### Merlin- and PI3K-docking protein Ezrin marks sites of lumen initiation.

**Fig 5.**
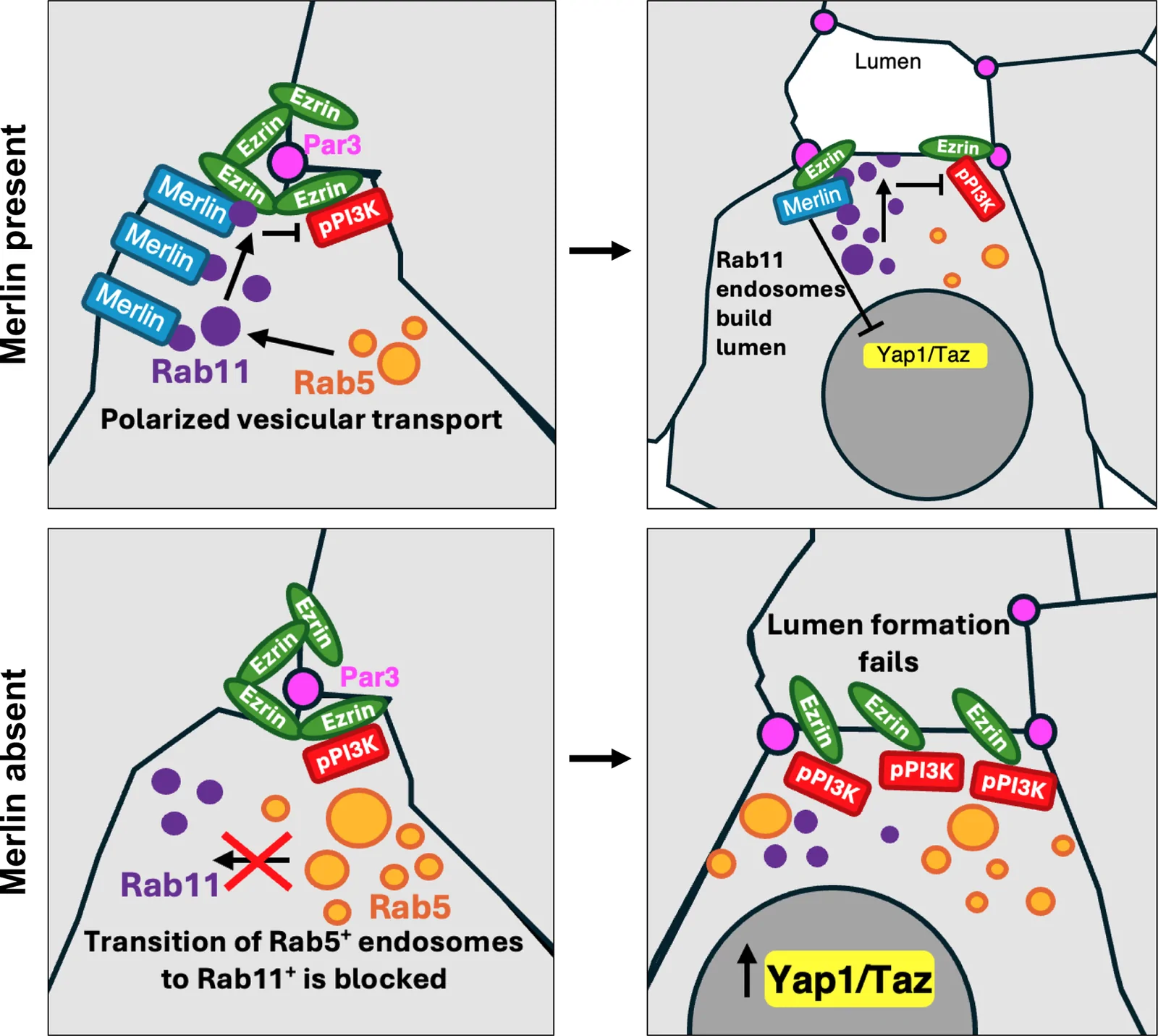
Model. Merlin establishes a spatial signaling domain that couples PI3K activity to apical membrane biogenesis. As AMIS formation begins, EZRIN is recruited first and MERLIN next. EZRIN helps recruit PI3K to the AMIS where it coordinates Rab5- and Rab11-mediated vesicular trafficking. MERLIN then restrains PI3K and YAP1/TAZ activity. In the absence of Merlin, elevated PI3K signaling disrupts apical membrane organization, leading to ectopic accumulation of apical determinants, impaired lumen morphogenesis, and increased Yap1/Taz activity. We postulate that excessive PI3K activity may alter endosomal maturation, favoring persistence of Rab5-positive early endosomal compartments at the expense of Rab11-positive recycling endosomes.^55–57^

Because both MERLIN and PI3K have been found to associate with the FERM domain-containing protein EZRIN (EZR),^45,46^ we asked whether EZR localizes to sites of lumen initiation, as it does in nascent lumen initiation in the kidney and human pluripotent stem cell (hPSC) cysts.^11,47^ In embryonic pancreatic epithelium, Ezrin was enriched at both AMIS and nascent microlumens (Fig. 4W–W”), and appears prior to Merlin at the AMIS, but is grossly co-localized at opening lumens. Of note, pPI3K is also localized to open lumens, but it marks distinct cortical regions of the apical membrane (**fig. S23**). This *in vivo* co-localization places Ezrin at sites where Merlin regulates lumen formation and interconnection of microlumens into a ductal plexus. Mechanistically, our data support a model in which Merlin coordinates apical membrane trafficking and epithelial morphogenesis by restraining Yap1/Taz and PI3K signaling at nascent lumens, potentially by competitively binding to Ezrin at the AMIS (Fig. 5).

### Merlin couples mechanical cues and intracellular trafficking.

Because Merlin functions as a mechanosensitive regulator in multiple tissues,^48,49^ we asked whether it responds to physical cues in pancreatic epithelial cells. Human pancreatic ductal epithelial cells (HPDECs) were cultured under sparse or confluent conditions to alter cell-cell contact and mechanical state. Under confluent conditions, MERLIN protein abundance was significantly increased (**fig. S22**). Rab5^+^ endosomal compartments were similarly increased. These data indicate both Merlin and Merlin-associated intracellular trafficking respond to changes in the physical environment experienced by pancreatic epithelial cells. Together with our developmental analyses, these findings raise the possibility that MERLIN links mechanical cues to apical trafficking and epithelial morphogenesis during formation of the pancreas.

## Discussion

This study identifies Merlin/Nf2 as a previously unrecognized mechanosensitive regulator of pancreas morphogenesis that couples epithelial architecture to lineage specification, including insulin-producing β-cells. Notably, Merlin is required during a restricted developmental window corresponding to lumen and plexus formation but is dispensable for early bud formation and maintenance of the pancreas. Loss of Merlin impairs *de novo* lumen formation, resulting in defective epithelial remodeling, altered lineage allocation, and reduced endocrine mass. Rather than functioning solely through canonical Hippo signaling, Merlin coordinates a broader signaling network that integrates PI3K signaling, polarity-directed vesicular trafficking, and epithelial cell behavior. These findings support a model in which pancreatic differentiation emerges from a mechanically organized epithelial state and suggest that Merlin establishes the tissue architecture needed for faithful developmental cell fate decisions.

### Epithelial morphogenesis as an instructive regulator of lineage allocation.

Across developmental systems, cell fate is determined not only by cell-intrinsic programs but also by the spatial organization of local niches, which impact cells non-autonomously. For instance in the Drosophila germarium, oocyte specification depends on the stereotypical geometry of a 16-cell cyst, with a single connected germ cell that adopts the oocyte fate.^50^ In mammalian skin, the position of a stem cell within the extended hair follicle niche predicts whether it remains quiescent or differentiates.^51^ We have shown that the pancreas represents a similar example, where the transient embryonic epithelial plexus serves as a morphogenetic niche and disruption of its lumens alters endocrine mass, including β-cells.^4,6,36,52^ When the plexus is blocked from resolving properly, as in the *Pdx1*^*Cre*^*;Afadin*^*f/f*^*;RhoA*^*f/f*^ mutant pancreas, endocrine progenitors incubate in this niche days longer and endocrine mass is tripled.^4^ By contrast, in the *Nf2*^*PancKO*^ pancreas, formation of the epithelial plexus is impaired, resulting in a persistent stratified epithelial state. In the early wildtype stratified pancreas, glucagon-positive cells can differentiate, but insulin-positive cells are rare. Consistent with this, we observe that glucagon-positive cells form relatively normally, whereas insulin-positive cells are markedly reduced in adult mutant pancreata. Our findings hence support the idea that tissue architecture itself is an instructive determinant of cell fate. Importantly, mosaic loss of Merlin does not alter lineage allocation, demonstrating that the differentiation defects observed in *Nf2*^*PancKO*^ pancreata arise from disruption of tissue architecture rather than cell-autonomous changes in fate programs. These findings support a model in which the pancreatic plexus acts as an instructive morphogenetic niche whose organization governs lineage output.

A notable implication of our findings is that transient morphogenetic intermediates can exert lasting effects on organ composition. The pancreatic plexus exists only briefly during development, yet perturbations that prolong or prevent its formation profoundly alter endocrine output with consequences that persist into adulthood. These observations suggest that developmental architectures may function as temporal regulatory niches that control lineage allocation before disappearing from the mature organ. More broadly, transient morphogenetic structures in other developing tissues may similarly act as instructive intermediates that shape adult tissue composition and function.

This work also has implications for understanding how endocrine mass is established. Current models emphasize transcriptional and growth factor signaling as primary determinants of endocrine differentiation. Our data suggest that tissue architecture constitutes an additional layer of developmental control: that of an instructive cellular neighborhood. Because endocrine cell mass that is established during development influences adult glucose homeostasis, transient defects in epithelial organization may have lasting physiological consequences long after morphogenesis has ended.

### Merlin functions beyond Hippo to restrain PI3K in pancreas.

Our findings support the idea that Merlin regulates signaling pathways beyond the Mst/Lats/Yap1/Taz axis. The incomplete rescue obtained by Yap1/Taz reduction further suggests that key functions of Merlin are separate from canonical Hippo signaling. Instead, our data supports a model in which Merlin regulates apical membrane biogenesis by spatially constraining PI3K-dependent trafficking pathways during epithelial lumen formation. Merlin localizes to membrane-associated polarity and cytoskeletal complexes that include Par3, Crb3 and Ezrin, positioning it to spatially coordinate signaling at the apical cortex. Prior studies have demonstrated that Merlin can directly suppress PI3K activity through interaction with the PI3K activator PIKE-L,^42^ while Ezrin and related cortex scaffolds are known to promote PI3K/Akt signaling at the plasma membrane.^45^ Because PI3K signaling strongly influences phosphoinositide composition, membrane identity and vesicle docking,^53^ aberrant PI3K activation following Merlin loss could disrupt the polarized trafficking machinery required for apical membrane biogenesis. Consistent with this model, loss of Merlin disrupts localization of Crb3-, Rab11- and Rab5-associated trafficking machinery and impairs lumen formation. We speculate that Merlin normally constrains PI3K signaling within apical cortical domains to ensure productive targeting and fusion of apical vesicles during lumenogenesis. Loss of this spatial control may lead to inefficient or ectopic vesicle delivery, impaired AMIS assembly and defective epithelial organization during pancreas development.

### Merlin interprets dynamic cell tension environments in pancreatic cells.

Changes in MERLIN expression and subcellular localization with epithelial cell density support emerging models positioning Merlin as a sensor of tissue mechanics.^49^ Recent studies showing assembly of Merlin and Hippo pathway components into membrane-associated cortical signaling domains provide a framework for understanding how mechanical information is translated into morphogenetic behaviors. The density-dependent changes we observe suggest that Merlin functions as part of a mechanically responsive cortical signaling network that integrates dynamic epithelial architecture with polarity, trafficking, and developmental signaling. In the growing pancreas, Merlin may dynamically interpret local epithelial cell density and tissue geometry to spatially coordinate lumen formation and lineage allocation during organ growth.

Recent studies provide a mechanistic framework for these observations by demonstrating assembly of Merlin and Hippo pathway components at the cell cortex.^54^ Rather than functioning solely as an upstream regulator of Hippo signaling, Merlin may organize spatially restricted signaling domains that integrate mechanics, polarity, vesicular trafficking, and growth control. Such a model may explain why polarity proteins, Rab-dependent trafficking pathways, PI3K signaling components, and Hippo regulators converge during pancreatic lumenogenesis. We speculate that Merlin-dependent condensates may act as mechanically responsive organizing centers that coordinate localization of Crb3-, Rab11-, Ezrin-, and PI3K-associated complexes, thereby spatially constraining signaling and membrane trafficking during AMIS formation and lumen fusion. Disruption of these signaling domains could impair apical trafficking and lumen organization, altering pancreatic lineage induction within the epithelial niche and ultimately reducing beta cell mass. More broadly, our findings support the concept that transient developmental architectures function as instructive intermediates that determine the cellular composition of mature organs.

## Materials and Methods

### METHODS

#### Ethics statement

All animal experiments were performed in accordance with the Guide for the Care and Use of Laboratory Animals and the Animal Welfare Act, as well as protocols approved by the University of Texas Southwestern Medical Center Institutional Animal Care and Use Committee (IACUC; approval number 2017–102243, approval date 26 September 2017). All animals were observed daily, and appropriate care was provided by the veterinary staff of the UTSW Animal Resource Center, which is fully accredited by the Association for Assessment and Accreditation of Laboratory Care, International (Unit Number 000673) and by the NIH Office of Laboratory Animal Welfare (Assurance Number D16–00296). Dams were euthanized via IACUC-approved humane methods, using carbon dioxide asphyxiation and secondary cervical dislocation.

#### Mice and embryo handling

CD1 mice were used as wild type controls. *Nf2*^*ff*^, *Pdx1*^*Cre-early*^, and *R26-tdTomato*^+/+^ were used for experiments in this study. For mosaic deletion experiments, Inducible deletion of Merlin was carried out by gavage of tamoxifen (tmx) (Sigma Aldrich, St. Louis, MO) at a dose of 75 mg/kg body weight to pregnant dams between E8.5 and E9.5. E11.5–E18.5 embryos were collected and dissected in PBS buffer.

Pancreata from mutant embryos were compared to WT or heterozygous tissue from littermate embryos. Genotypes were determined by PCR after O/N digestion using DirectPCR (Tail) Lysis buffer (Viagen, Los Angeles, CA) per manufacturer’s instructions. Briefly, inducible simultaneous deletions were obtained by mating *Nf2*^*f/f*^ females with *Nf2*^*f/+*^*;Pdx1*^*Cre*^ males. To obtain mosaic *Nf2*-deficient clones, pregnant dams were gavaged with 0.75 mg/kg tmx at E9.5. Tamoxifen induction was performed as previously described.^36^

#### Generation of myrTom mice

N-myristoylated tdTomato Lox-Stop-Lox (Myr-tdTom-LSL) mice were generated by homologous recombination using the genomic ROSA26 locus. N-myristoylated tdTomato was generated by PCR from pCA-mTmG (a gift of Liqun Luo, Addgene # 26123) using primers that added 5’ and 3’ Mlu1 sites. The PCR product was ligated in Ai9 (a gift from Hongkui Zeng, Addgene #22799) using Mlu-1 site. The targeting vector was linearized by Kpn1 and then transfected into mouse embryonic stem cell line SM-1 using the UTSW Transgenic Core facility. A target allele containing the Neo-resistance cassette (Neo^r^) were selected with geneticin (G418) and diphtheria toxin A (DTA). Targeted clones were screened by long-range PCR of extracted genomic DNA, with correctly-targeted clones producing a 3.2 kb product from the 5’ arm and a 4.6kb from the downstream 3’arm. The primer sets utilized are the following: 5’arm PCR: 5’-GACTAGGGCTGCGTGAGTCTC-3’/5’-AGAAGAAGGCATGAACATGGTTAG-3’; and 3’arm PCR: 5’-CAGCAGCCTCTGTTCCACATAC-3’/5’-TGCAGTGTTGAGGGCAATCTG-3’.

For further verification, PCR was also performed for Neo^r^, loxP, and Myr-tdTomato. Three correctly-targeted ES cells (3B2, 4E11, 4H3) were injected into blastocysts obtained from C57/Bl6 females. The 4H3 clone was used for all experiments.

#### Sectioning

Post dissection, pancreata were fixed in 4% PFA at 4°C overnight. For paraffin sectioning, tissues were gradually dehydrated to 100% ethanol, then rinsed twice in 100% ethanol, twice in xylene for 30 min at room temperature, a mixture of 1:1 paraplast:xylene for 10 min at 60°C, and then a series of 100% paraplast at 60°C (McCormick Scientific). The tissues were then embedded in paraplast and sectioned at 10 μm with a Biocut 2030 microtome. SuperfrostPlus glass slides (Fisher) were used. For cryosectioning, tissues were rinsed in PBS and incubated in 30 % sucrose overnight at 4°C for cryoprotection. The next day, the tissues were rinsed in OCT twice for 30 min each at room temperature. The tissues were embedded in OCT, snap-frozen on dry ice, and sectioned at 30 μm using a Leica CM-3050S cryostat. SuperfrostPlus glass slides (Fisher) were used.

#### Immunostaining on sections

Paraffin sections were deparaffinized with xylene, rehydrated through ethanol wash series into PBS, permeabilized in PBS + 0.3% Triton X-100 for 10 min, and then treated with R buffer A (nuclear antigens) or R buffer B (cytoplasmic antigens) in a 2100 Retriever (Electron Microscopy Sciences). Slides were incubated in CAS Block (Invitrogen) in PBS for at least 1 hour and then in primary antibody overnight at 4°C, washed in PBS, and then incubated in secondary antibody for 2 h at room temperature. Slides were then washed again in PBS and mounted using Prolong Gold (with or without DAPI). For nuclear staining, slides were also incubated in DAPI in PBS for 10 min at room temperature prior to mounting. For cryosections, slides were baked for 30 min at 60°C, rinsed in PBS, and treated for antigen retrieval as above. For cryosections, all washes were performed in PBS. Slides were permeabilized in 0.3% Triton X-100 in PBS for 10 min, blocked in CAS-Block, and incubated in primary antibody overnight at 4°C. The next day, slides were washed, incubated in secondary antibody for 2 h at room temperature, and washed again. Mounting and nuclear staining were performed as above.

For both paraffin and cryosections, images were acquired either on the Nikon CSU-W1 SoRa confocal microscope at the Quantitative Light Microscopy Core, or the Nixon-AXR or Nikon A1R confocal provided by the UT-Southwestern Molecular Biology department.

#### Whole-mount immunostaining

For embryonic tissues, immunostaining was carried out as described before.^52^ For postnatal pancreata, tissues were washed in PBS following fixation and stored in PBS overnight at 4°C. Next, tissues were dehydrated into 50% methanol, incubated in 50% methanol for 1 h at room temperature, rehydrated back into PBS, and permeabilized in PBS + 1% Triton X-100 for 2 h at room temperature. Tissues were blocked in CAS-Block for at least 2 h and incubated in primary antibody overnight at 4°C. Tissues were then washed in PBS six times for 1 h each, incubated in secondary antibody overnight at 4°C, and washed in PBS five times for 30 min each. Next, tissues were dehydrated to 100% methanol, washed three times, and mounted in BABB (one-third benzyl alcohol and two-thirds benzyl benzoate) on slides using coverslip spacers. Images were acquired either on a Zeiss LSM710 Meta confocal, or Nikon1-AXR provided by the UT-Southwestern Molecular biology department.

##### Light sheet microscopy:

Pancreata for light sheet microscopy were processed as above. After secondary antibody washes, pancreata were mounted in low-melting agarose and then gradually dehydrated to 100% methanol. At least 3 washes with methanol were carried out to ensure complete dehydration of agarose block and then blocks were cleared with BABB. Agarose blocks were secured to glass slides and imaged using cleared-tissue axially swept light-sheet microscopy (ctASLM), as previously described.^55^ Briefly, samples were imaged on a custom ctASLM microscope equipped with matched cleared-tissue illumination and detection objectives (54-10-12, Applied Scientific Instrumentation), four laser excitation lines (LX 405–100C, LX 488–50C, LS 561–50 and LX 637–140C, Coherent), and sCMOS camera detection (ORCA-Flash 4.0 v2, Hamamatsu). Images were acquired with a 737 × 737 μm field of view, with raw lateral and axial resolutions of 0.83 μm and 0.94 μm, respectively, and deconvolved resolutions of 0.62 μm and 0.72 μm, respectively.

#### Vibratome sectioning and immunostaining

After dissection and fixation, tissue was embedded in 2.5–5% low melting point agarose (Invitrogen). 200 μm sections were cut using a Leica vibratome. Floating sections were permeabilized with Triton X-100 in PBS (1% for 1.5 h at room temperature. Sections were blocked in CAS-Block for 1 h and incubated in primary antibody mixture overnight at 4° with gentle nutation. Sections were washed in PBS (5 × 1 h) and were incubated in secondary antibody mixture overnight at 4°. Sections were washed in PBS (5 × 30 min). Sections were placed on Superfrost Plus glass slides and cleared in a bubble of Rapiclear 1.47 (Cedarlane Labs) for at least 10 min. Slides were mounted in Rapiclear and imaged as in previous sections.

#### Glucose Tolerance Test

Intraperitoneal glucose tolerance tests were performed in mice fasted overnight. Baseline blood glucose levels were measured from tail vein blood using a handheld TRUEbalance glucometer. Glucose (2 g/kg body weight) was administered by intraperitoneal injection, and blood glucose levels were measured at 15, 30, 60, 90, and 120 min post-injection. Data were analyzed by 2-way ANOVA. Area under the curve (AUC) was calculated using GraphPad Prism.

#### Dry Weight of Pancreas

Adult mice (~18 weeks of age) were euthanized and pancreata were dissected. Adipose tissue was removed, and pancreata were briefly blotted to remove surface moisture prior to weighing on an analytical balance. Pancreatic weights were recorded in Microsoft Excel.

#### qPCR

Real-time qPCR was performed as described previously.^52^ Briefly, 500ng of total RNA was isolated from individual E14.5 mouse pancreata (dissected on ice) using RNeasy Microkit (Qiagen). cDNA was synthesized using SuperScript III (Invitrogen). 1μl of cDNA in Power SYBR Green Master mix (Applied Biosystems) was used for real-time qPCR analysis (QuantStudio3, ThermoFisher) of gene expression. Gene expression levels were normalized to *GAPDH* of littermate control, and the ΔΔC_t_ method was used to calculate fold change. Data were collected from individual embryos (*n* = 5 per genotype), and samples were analyzed in triplicate. Data are presented as mean (SD).

#### Single nuclear RNA sequencing

##### Nuclear Isolation:

For E11.5 samples, pancreata were dissected from five litters, and all pancreatic buds collected between E11.25 and E11.75 were pooled, yielding approximately 40 pancreatic buds in total. Samples were snap-frozen in liquid nitrogen and stored at −80°C until processing. For E14.5 samples, individual pancreata were collected from three control (Cre-negative, littermate) and three *Nf2*^*PancKO*^ embryos, snap-frozen in liquid nitrogen, and stored at −80°C until processing. Nuclei were isolated from frozen tissue using the 10x Genomics Chromium Nuclei Isolation Kit according to the manufacturer’s protocol. Tissues were lysed on ice for 5 minutes prior to nuclei purification. For E14.5 samples, nuclei from pancreata of the same genotype were pooled before library preparation. Single-nucleus RNA-sequencing libraries were generated using the 10x Genomics Next GEM Single Cell 3′ Reagent Kit v3.1 and sequenced at the UT Southwestern Next Generation Sequencing Core on an Illumina NextSeq 500 High Output Flow Cell using v2.5 chemistry. Low-quality nuclei and potential doublets were removed during quality control.

##### RNAseq data analysis:

Single-nucleus RNA-seq data were processed using standard quality-control, normalization, clustering, trajectory inference, and differential expression workflows as described in the Supplementary Methods.

##### Code Availability:

Custom software used for diffusion map analysis, time-embedded diffusion integration, geometric harmonics projection, EIGEN marker identification, and downstream analyses is available at: https://github.com/cpchaney/pdx1-cre_nf2

#### Pancreatic sphere assay and immunostaining

##### Sphere formation:

Sphere assays were carried out by isolating E12.5 pancreata, dissociating pancreata into single cells and culturing in Matrigel as previously described.^35^ Dispase treatment and subsequent mesenchyme removal in PBS were each performed in a small drop of liquid in a regular dissection dish. The pancreata were transferred into 100 μL of TrypLE using forceps, and 500 μL of DMEM + FBS was used to stop trypsinization. Cell clumps were broken by pipetting up and down, and cells were centrifuged at 300g for 5 min. Cells were then resuspended in an appropriate volume of culture medium described in the original protocol. The Matrigel–cell mix was seeded on coverslips in coverglass-bottom plates. Spheres were fixed after 3 days in culture. Spheres were cultured in PEM media (DMEM F12, 10% FBS, 64ng/mL FGF2, and 10 μM Y-27632, gentamycin).

##### Immunostaining:

Immunostaining was performed as follows: Spheres were rinsed with PBS on ice and fixed in 4% PFA in PBS for 10 min at room temperature. Next, spheres were washed in PBS + 0.1% NP40 (PBSN) and permeabilized in PBSN for 15 min at room temperature. Spheres were blocked in CAS-Block for 30 min, incubated in primary antibody overnight at 4°C, washed in PBS, incubated in secondary antibody for 1 h, washed in PBS again, and then imaged.

#### Explant Culture

Pancreatic explants were performed as previously described.^4^ In brief, E11.5-E12.0 pancreata were dissected from embryos, and placed on a fibronectin-coated, coverglass bottom 24 well plate. DMEM/F12, 10%FBS, +gentamycin media was utilized to culture pancreata. Media was changed every other day, unless otherwise noted for drug treatment. Explants were maintained at 37°C, 5% CO_2_.

##### Drug treatment:

Treatment with bpV(pic) and LY294002 was performed as previously described.^20^ For LY294002 experiments, dimethyl sulfoxide (DMSO) was used as the vehicle control. For bpV(pic) experiments, sterile distilled water was used as the vehicle control. Drugs were diluted to the appropriate concentrations in explant culture medium immediately prior to use. To administer treatments, existing medium was aspirated under sterile conditions and replaced with medium containing either vehicle or drug. For all drug treatment experiments, fresh treatment medium was prepared daily and replaced every 24 hours.

##### Immunofluorescence on explants:

Explants were fixed in 4% paraformaldehyde (PFA) for 10 minutes at room temperature and subsequently washed three times with PBS. Following fixation, explants were gently detached from the culture dish using forceps and transferred to glass vials. Explants were permeabilized in PBST (PBS containing 1% Triton X-100) for 1 hour, blocked in CAS-Block for 30 minutes to 1 hour, and incubated with primary antibodies overnight at 4°C. The following day, explants were washed three times with PBS, incubated with secondary antibodies for at least 1 hour at room temperature, and washed three additional times with PBS. Explants were mounted on glass slides using iSpacer tape (SunJin Lab Co.) and cleared in RapiClear prior to imaging.

##### Live imaging:

All live imaging was performed on a Nikon CSU-W1 SoRa confocal microscope at the Quantitative Light Microscopy Core at UT Southwestern Medical Center. For overnight imaging experiments, images were acquired at intervals of no greater than 10 minutes for a minimum imaging duration of 8 hours, depending on microscope availability. All dual-reporter-positive explants from each litter were imaged.

Because littermate controls were not always available (for example, a litter may contain a *Pdx1*^*Cre*^*; Nf2*^*ff*^*, myrTom*^*f/+*^*; Crb3*^*GFP+*^ mutant but no corresponding cre+ control embryos), stage-matched control explants from independent litters were used for comparison. For analysis of vesicular trafficking events, live imaging was performed at interval of 1 frame per 25 seconds (one z-stack), where a frame is defined as a complete z-stack at 0.5um intervals utilizing the 100x objective. Imaging was performed for approximately 10 minutes. All live imaging was done at 37°C with 5% CO_2_.

#### 2D cell culture

##### Primary cell isolation and culture:

Pancreata were dissected at E12.5, mesenchyme was manually removed by dissections with forceps post treatment with Dispase. Pancreatic buds were transferred to individual sterilized microcentrifuge tubes and dissociated using 100uL TrypLE. 500uL of DMEM/10% FBS was utilized to neutralize Trypsin. Cells were plated on a fibronectin-coated coverglass bottom plate and cultured in PEM media in standard tissue culture conditions (37°C with 5% CO_2_).

##### Human Pancreatic Ductal Epithelial Cell Culture:

A vial of human pancreatic ductal epithelial cells (HDPE-H6c7)^56^ were obtained from Kerafast and cultured in Keratinocyte Serum Free Media supplemented with bovine pituitary extract and EGF. Cells were cultured on gelatin-coated chamber slides at tissue culture conditions.

##### Immunofluorescence on cells:

Cells were fixed in 4% PFA for 10 minutes at room temperature. Cells were then washed three times in PBS, permeabilized for 10 minutes in PBSN (0.1% NP-40), and blocked in CAS block for at least 30 minutes. Cells were then incubated in primary antibodies overnight with gentle nutation at 4° C. The next day, cells were washed three times in PBS and incubated in secondary antibody for at least 1 hour. Cells were washed three times and mounted in either Fluoromount (HDPE cells) or imaged directly in the well.

#### Image Quantification

Quantification of lineage volume, epithelial plexus architecture, cell death, proliferation, and differentiation was performed using established image analysis pipelines;^3,4,6,36,52,57^ detailed methodologies and parameter settings are provided in the Supplementary Methods. Image analysis was performed blinded to genotype whenever feasible.

#### Statistical analysis

Statistical analyses were performed using GraphPad Prism version 10.5.0 (774; GraphPad Software, Inc.). Unless otherwise noted, statistical significance was assessed using Welch’s t-test. P-values are reported in the figure legends. Formal power analysis was not performed prior to data collection; however, sample sizes were chosen to be consistent with those reported in previously published studies in the field. No samples or animals were excluded from analysis unless predefined technical failure criteria were met.

## Supplementary Material

Supplementary Files

This is a list of supplementary files associated with this preprint. Click to download.

- MovieS3.mp4
- MovieS3.mp4
- MovieS3.mp4
- MovieS3.mp4
- MovieS12.mp4
- MovieS12.mp4
- MovieS12.mp4
- MovieS12.mp4
- MovieS13.mp4
- MovieS13.mp4
- MovieS13.mp4
- MovieS13.mp4
- MovieS9.mp4
- MovieS9.mp4
- MovieS9.mp4
- MovieS9.mp4
- MovieS7.mp4
- MovieS7.mp4
- MovieS7.mp4
- MovieS7.mp4
- MovieS10.mp4
- MovieS10.mp4
- MovieS10.mp4
- MovieS10.mp4
- MovieS1.mp4
- MovieS1.mp4
- MovieS1.mp4
- MovieS1.mp4
- MovieS11.mp4
- MovieS11.mp4
- MovieS11.mp4
- MovieS11.mp4
- MovieS6.mp4
- MovieS6.mp4
- MovieS6.mp4
- MovieS6.mp4
- MovieS16.mp4
- MovieS16.mp4
- MovieS16.mp4
- MovieS16.mp4
- MovieS5.mp4
- MovieS5.mp4
- MovieS5.mp4
- MovieS5.mp4
- SupplementalInfoTextNatureCommsF.pdf
- SupplementalInfoTextNatureCommsF.pdf
- SupplementalInfoTextNatureCommsF.pdf
- SupplementalInfoTextNatureCommsF.pdf
- MovieS4.mp4
- MovieS4.mp4
- MovieS4.mp4
- MovieS4.mp4
- MovieS8.mp4
- MovieS8.mp4
- MovieS8.mp4
- MovieS8.mp4
- MoviesS2.mp4
- MoviesS2.mp4
- MoviesS2.mp4
- MoviesS2.mp4
- SupplementalFigs.pdf
- SupplementalFigs.pdf
- SupplementalFigs.pdf
- SupplementalFigs.pdf
- MovieS14.mp4
- MovieS14.mp4
- MovieS14.mp4
- MovieS14.mp4
- MovieS17.mp4
- MovieS17.mp4
- MovieS17.mp4
- MovieS17.mp4
- MovieS15.mp4
- MovieS15.mp4
- MovieS15.mp4
- MovieS15.mp4
- MovieS18.mp4
- MovieS18.mp4
- MovieS18.mp4
- MovieS18.mp4

## HIGHLIGHTS

- A transient embryonic epithelial architecture is required to establish adult endocrine cell mass.
- Merlin-dependent lumenogenesis and plexus morphogenesis non-cell-autonomously regulate pancreatic lineage allocation.
- Merlin is required to coordinate vesicular trafficking during *de novo* lumenogenesis
- Merlin suppresses the Hippo and Pi3K pathways to facilitate lumen formation
